# Non-contact meibography changes according to disease activity in rheumatoid arthritis cases

**DOI:** 10.1186/s12886-023-03194-8

**Published:** 2023-11-13

**Authors:** Amr Mounir, Mohamed Anbar, Islam Awny, Tasneem Mohammed Bakheet, Ola Mounir, Elshimaa A Mateen Mossa

**Affiliations:** 1 Ophthalmology Department, Sohag Faculty of Medicine, Sohag University, Almohafza St., Sohag, Sohag City, 82511 Egypt; 2 Public Health and Community Medicine Department, Faculty of Medicine, Sohag University, Sohag City, Egypt; 3 Rheumatology and Rehabilitation Department, Faculty of Medicine, Sohag University, Sohag City, Egypt

**Keywords:** Meibography, Rheumatoid Arthritis, Mebioscore, Scheimpflug topographer

## Abstract

**Purpose:**

To measure dry eye disease (DED) activity in rheumatoid arthritis (RA) patients, correlate it with the activity and duration of RA, and objectively measure the degree extent of DED in RA active cases.

**Methods:**

The paper studied the meibomian gland of 30 RA cases referred by the Rheumatology Department, Sohag University Hospitals to Sohag Cornea and Refractive Center, Sohag, Egypt, by infrared non-contact meibography in the Scheimpflug topographer (Sirius, CSO, Italy) from July 2021 to July 2022. The cases fulfilled the RA classification criteria according to the 2010 American College of Rheumatology and European League and underwent full lab investigations. They were distributed to two groups based on the DAS-28 questionnaire. The patients were distributed into low to moderate activity (3.2 < score <  = 5.1) and high activity groups (score > 5.1).

**Results:**

This study included 60 eyes of 30 RA patients. They scored a mean age of (44 ± 10 years), number of swollen joints (3 ± 3), number of tender joints (5 ± 3), duration of disease (8 ± 4), activity of the disease measured by DAS-28 (4.4 ± 0.9), and sex (males were 9.7% vs females 90.3%). The number of swollen joints, tender joints, ESR and DAS-28 were more in the high activity group with the *p*-value of (0.018, 0.001, < 0.003 and < 0.004), respectively. There are no statistically significant differences between both groups as regards disease duration (*p*-value of 3.8). The high activity group showed significant affection regarding the mebioscore of the lower and upper lids, total mebioscore, percentage of meibomian glands in upper and lower lids, first non-invasive break-up time test (NIV-BUT) of the tear film, as well as average non-invasive break-up time test (NIavg-BUT). There were moderate correlations between the activity of the disease represented by (DAS-28) and different parameters evaluating eye dryness (meiboscore of the lower and upper lids, total meiboscore, percentage of meibomian glands in upper and lower lids, NIV-BUT of the tear film, and NIavg-BUT of the tear film).

**Conclusion:**

There was no correlation with duration of RA but moderate correlations between the activity of the disease represented by (DAS-28) and different parameters evaluating eye dryness in RA patients with valuable use of noncontact Meibography to evaluate eye dryness in RA cases. Both Meiboscore and Meiboscale of Non-contact Meibography were found to be useful tools in grading of dry eye disease in different activity grades of Rheumatoid Arthritis.

## Introduction

Dry eye disease (DED) is one of the common annoying diseases that influences the ocular surface and the tear film. DED could be manifested by visual disturbance, eye discomfort, and tear film instability, which leads to damage of the ocular surface [1].

Several systemic conditions is associated with DED, including immunologic diseases, degenerative diseases, metabolic diseases, cardiovascular disorders and malignancies [2].

RA is one of the most common immunological diseases associated with DED, it is a type of inflammatory arthritis, which denotes a systemic chronic autoimmune condition that causes joint synovitis, as well as multiple symmetric small and large joint lesions [3–5]. It affects female patients 2–3 times more than males and happens in people aged 40–60 years [6, 7].

*Most previous studies based on the population assessed DED using a questionnaire but did not carry out an objective ocular examination *[8].

Complicated gene-environment interactions causes destruction of immune tolerance and a typical symmetrical pattern of synovitis presents [9]. In some cases, a set of autoantibodies activating the complement pathways, known as the rheumatoid factor (RF), causes extra-articular damage of them DED with the pathogenesis of mucosal autoimmune disease [10–13].

This study aimed to objective measurement of DED activity in RA cases and its correlation to disease activity.

### Patients and methods

The study concerned with assessment of meibomian gland of 30 RA active cases referred by the Rheumatology Department, Sohag University Hospitals to the Sohag Cornea and Refractive Center, Sohag, Egypt, by infrared noncontact meibography in the Scheimpflug topographer (Sirius, CSO, Italy) from July 2021 to July 2022. The cases met the RA classification criteria of the 2010 American College of Rheumatology and European League [14] and underwent full lab investigations.

Exclusion criteria included dry eye-related diseases other than RA, such as pemphigus vulgaris, sarcoidosis, Steven Johnson infections, e.g., HCV (hepatitis C virus) and HIV (human immunodeficiency virus), MD, contact lens wear, refractive corneal surgery history, using antidepressants and parasympatholytic drugs, as well as other ophthalmic disorders, particularly blepharitis, or long-lasting use of Eye drops containing preservatives for 3 months or more before examinations.

This study was approved by the Ethical Committee of the Faculty of Medicine, Sohag University, under IRB registration number: *Soh-Med-22–10-25* and carried out following the Helsinki declaration. All participants provided informed consent before the clinical assessments.

### Rheumatological assessments

Assessments of RA cases included full history, such as disease duration, full physical examinations, focusing on joint examination. Assessing the RA disease activity was applied to all cases based on DAS-28, which included 28 tender joints, 28 swollen joints, erythrocyte sedimentation rate, and using the visual analog scale for the general health assessments. The disease activity scores were as follows: High (> 5.1), moderate (> 3.2 to ≤ 5.1), and mild (3.2 or less). A DAS-28 below 2.6 corresponded with being in remission according to the American Rheumatism Association criteria [15]. Using the DAS-28 questionnaire, the patients were distributed into two groups according to the second DAS-28 grading system, They were divided into a low to moderate activity group (= 2.6 < score <  = 5.1) and high activity group (score > 5.1) [16].

Lab examinations were the complete blood count (CBC), C-Reactive protein (CRP), erythrocyte sedimentation rate (ESR) based on the quantitative Westergen’s method (mm/h), liver and kidney functions, rheumatoid factor (RF) based on the latex agglutination test supplied by Spin React, anti-cyclic-citrullinated peptide (Anti CCP), anti-SSB/La, and anti-SSA/Ro antibodies tested by ELISA technique (AlegriaVR assay provided by Orgentec Diagnostika GmbH, Germany).

### Ophthalmologic examination

Both eyes of each patient were tested. The current research studied the meibomian gland of 30 RA cases referred by the Rheumatology Department, Sohag University Hospitals to the Sohag Cornea and Refractive Center, Sohag, Egypt, by infrared noncontact meibography in the Scheimpflug topographer (Sirius, CSO, Italy) from July 2021 to July 2022.

### Assessment

#### Non-invasive measurements of tear film break-up dynamics

The tear film break-up dynamics were assessed in a dark room, and the participants were instructed to blink in a natural manner two times, then make the eyes open for ten seconds or more. Immediately after blinking, the Scheimpflug-Placido topographer (Sirius, CSO, Firenze, Italy) was used to record the tear film break-up video for ten seconds. Simultaneously, the Placido disc pattern (22 black and white rings) was projected to the cornea using the device. The times and locations of tear break-up spots were restructured into a 2D precorneal tear film map. This measurement was performed three times for each eye with a one-minute interval at least after each measurement. Moreover, the participants were instructed to blink naturally.

#### Non-contact Meibography

The Sirius (CSO, Florence, Italy) corneal topographic device with the Phoenix-Meibography Imaging software module was utilized to perform the non-contact meibography. Cases were seated facing the scanner, and their forehead was in contact with the headrest. The upper eyelid only was everted and assessed as being a better inter-examiner agreement as regards grading as it contains meibomian glands MGs more than that of the lower eyelid and are longer [17, 18].

“Dropout” represented MGs that did not transverse the total tarsal plate. The Phoenix software provided the dropout measurement in percentages and assembled the dropout by a scale in the area indicated by the user’s free-hand tool: 4 (> 75%), 3 (51–75%), 2 (26–50%), 1 (≤ 25%), and 0 (no loss) [6].

### Statistical analysis

SPSS (V. 25.0) was utilized for data analysis. While quantitative data were given as means ± standard deviation, the qualitative ones were given as numbers and percentages. Independent sample T-tests were utilized for making a comparison of the eye dryness parameters between the two groups. A 5% level was selected as a significant level in all statistical tests. Also, bivariate correlation analysis was done to detect the correlation between DAS score as an indicator for the disease activity and eye dryness parameters.

## Results

The research population comprised 60 eyes of 30 participants who had rheumatoid arthritis (RA). They had a mean age of (44 ± 10 years), number of swollen joints (3 ± 3), number of tender joints (5 ± 3), duration of disease (8 ± 4), disease activity assessed by DAS-28 (4.4 ± 0.9), and sex (9.7% males were vs 90.3% females), see Table 1.

**Table 1:** The population’s clinical and demographic data (*n* = 30)

		**Low to moderate** ***N* = 22**			**High activity** ***N* = 8**			***P* value**
**Age**		**43.27**	** + **	**9.94**	**46.5**	** + **	**8.28**	**0.65**
**Duration of disease (years)**		**7.6**	** + **	**3.8**	**9.1**	** + **	**4.2**	**0.77**
**Number of swollen joints**		**3**	** + **	**2**	**5**	** + **	**3**	**0.018**
**Number of tender joints**		**4**	** + **	**2**	**7**	** + **	**1**	**0.001**
**DAS-28**		**4.1**	** + **	**.7**	**5.4**	** + **	**.2**	** < 0.004**
**Sex** **No. (%)**	**Female**	**19**		**86.4%**	**8**		**100%**	**0.2**
	**Male**	**3**		**13.6%**	**0**		**0%**	
**ESR**		**42**	** + **	**21**	**81**	** + **	**11**	** < 0.003**
**Anti-Ro**		**Negative**			**Negative**			**–––-**
**Anti-La**		**Negative**			**Negative**			**–––-**
**ALT**		**19**	** + **	**7**	**19**	** + **	**7**	**0.87**
**S. Creatinine**		**.807**	** + **	**.133**	**.825**	** + **	**.139**	**0.75**
**ANTI-CCP**		**154.25**	** + **	**94.62**	**199.15**	** + **	**115.30**	**0.28**
**CRP**	**positive**	**17**		**77.3%**	**6**		**75.0%**	**0.89**
	**negative**	**5**		**22.7%**	**2**		**25.0%**	
**RF**	**Positive**	**18**		**81.8%**	**5**		**62.5%**	**0.26**
	**Negative**	**4**		**18.2%**	**3**		**37.5%**	

DAS-28 (disease activity score) DAS-28, ALT, ESR, WBCs, HG, ESR, ALT, CRP, S. Creatinine, Anti-CCP, CRP, RF, *P*-value were computed using the independent sample T-Test or Chi-Square test wherever suitable

These cases were distributed into two groups based on the DAS-28 questionnaire. They were divided into low to moderate activity group (= 2.6 < score <  = 5.1) and high activity group (score > 5.1). Twenty-two patients (22) had low to moderate activity, and eight (8) patients had high activity. The groups were comparable in the mean age (43.27 ± 9.94 vs 46.5 ± 8.28 years, *p*-value = 4.2) and sex (females were 19% vs 8 and males were 3% vs 0%, *p*-value = 0.2) for low to moderate activity and high activity groups, respectively.

There are no statistically significant differences between both groups regarding disease duration (*p*-value of 3.8).

The number of swollen joints, tender joints, and DAS-28 were more in the high activity group that had the *p*-value of (0.018, 0.001, and < 0.004), respectively.

Different laboratory parameters were compared in both groups in (Table 2). The erythrocyte sedimentation rate (ESR) was higher for the high activity with a *p*-value (< 0.003).

**Table 2:** Comparison between the two groups as regard demographic and clinical data and laboratory data, *n* = 30

		**Mean**	** + **	**SD**
**Age**		**44**	** + **	**10**
**Number of swollen joints**		**3**	** + **	**3**
**Number of tender joints**		**5**	** + **	**3**
**Duration of disease**		**8**	** + **	**4**
**DAS-28**		**4.4**	** + **	**.9**
**Gender** **No. (%)**	**Females**	**28 (90.3%)**		
	**Males**	**3 (9.7%)**		

High activity group showed significant affection regarding the mebioscore of the lower and upper lids, total mebioscore, percentage of meibomian glands in upper and lower lids, first non-invasive break-up time test (NIV-BUT) of the tear film, as well as average non-invasive break-up time test (NIavg-BUT) of the tear film, as shown in (Table 3).

**Table 3:** Assessment of eye dryness among the two studied groups, *n* = 30

	**Low to moderate**			**High activity**			*P value
	Mean	+	SD	Mean	+	SD	
	Median (IQR)			Median (IQR)			
Meiboscore of the upper lid	1.36	+	0.7	2.31	+	0.64	0.011
	1 (1–2)			2 (2–2.5)			
Meiboscore of the lower lid	0.73	+	.55	1.63	+	0.52	0.001
	1(0–1)			2(1–2)			
Total meiboscore	2.09	+	0.87	3.5	+	0.76	0.001
	2 (1–3)			4(3–4)			
NIV-BUT	6.7 ± 3.2 6.1(4.4 _8.8)			1.5 ± 0.9 1.2(1–1.3)			< 0.005
NIavg-BUT	9.8 ± 1.8 10 (8.2–10.5)			3.5 ± 2.00 2.7 (2–5.5)			< 0.003
MGL % UL	46.41 ± 10.52 45 (40–50)			14.38 ± 6.78 15(12.5–20)			< 0.003
MGL % LL	29.55 ± 9.37 30 (25–35)			8.5 ± 6.12 9.5(3.5–11.5)			< 0.001

NIV-BUT, NIavg-BUT, MGL % UL (meibomian gland percentage in the upper lid), MGL % LL (meibomian gland percentage in the lower lid), *P*-value was computed by the Mann–Whitney U

**Fig. 1:**
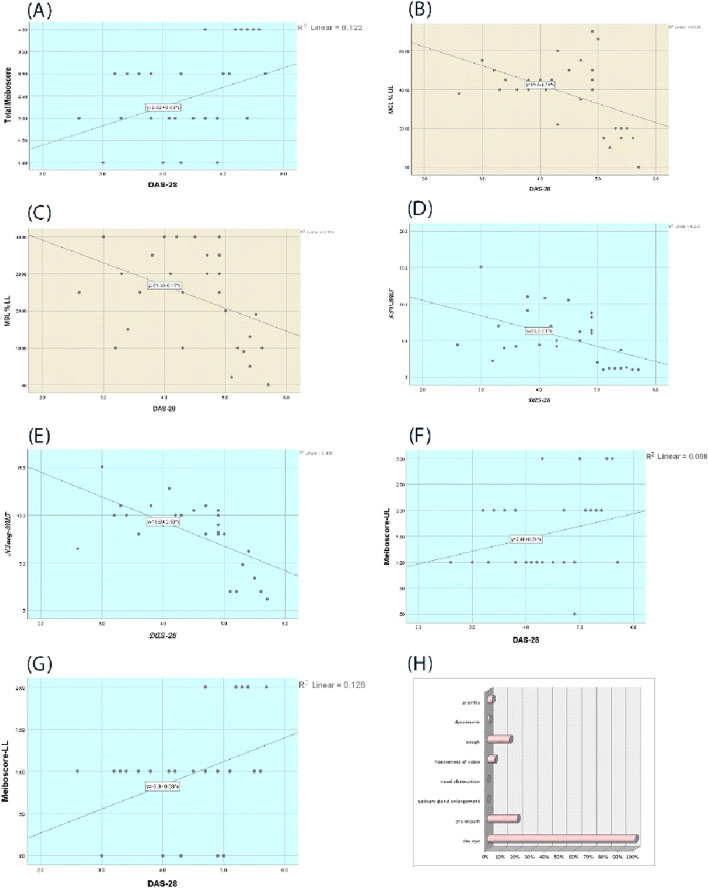
**A** Correlation between DAS-28 and total Meiboscore, **B** Correlation between DAS-28 and MGL % UL **C** Correlation between DAS-28 and MGL % LL correlation, **D** Correlation between DAS-28 and non-invasive breakup time test. **E** Correlation between DAS-28 and average non-invasive average breakup time test **F**) Correlation between DAS-28 and Meiboscore-UL. **G** Correlation between DAS-28 and Meiboscore-LL, H) The percentage of non-ophthalmological manifestations in studied rheumatoid arthritis patients

There were moderate correlations between the activity of the disease represented by (DAS-28) and different parameters evaluating eye dryness, There were positive moderate correlation between (DAS-28) and Meiboscore of the lower and upper lids, total Meiboscore. While there were negative moderate correlations between (DAS-28) and percentage of meibomian glands in upper and lower lids, first NIV-BUT of the tear film, and NIavg-BUT of the tear film, as shown in Table 4 and Fig. 1A: G.

**Table 4:** Bivariate correlation between DAS-28 and different parameters evaluating eye dryness

	R	P
Total Meiboscore	0.4	0.05
Meiboscore of the upper lid	0.26	0.1
Meiboscore of the Lower lid	0.4	0.05
NIV-BUT	-0.5	0.006
NIavg-BUT	-0.64	< 0.001
MGL % UL	-0.5	0.008
MGL % LL	-0.4	0.02

NIV-BUT (first non-invasive breakup time test), NIavg-BUT (average non-invasive break-up time test), MGL % UL (meibomian gland percentage in the upper lid), MGL % LL (meibomian gland percentage in the lower lid), and DAS-28 (disease activity score)

RA affects many parts of the body, with the most prevalent affection targeting the joints and the eye. The percentage of non-ophthalmological manifestations in studied rheumatoid arthritis patients is summarized in Fig. 1H.

**Fig. 2:**
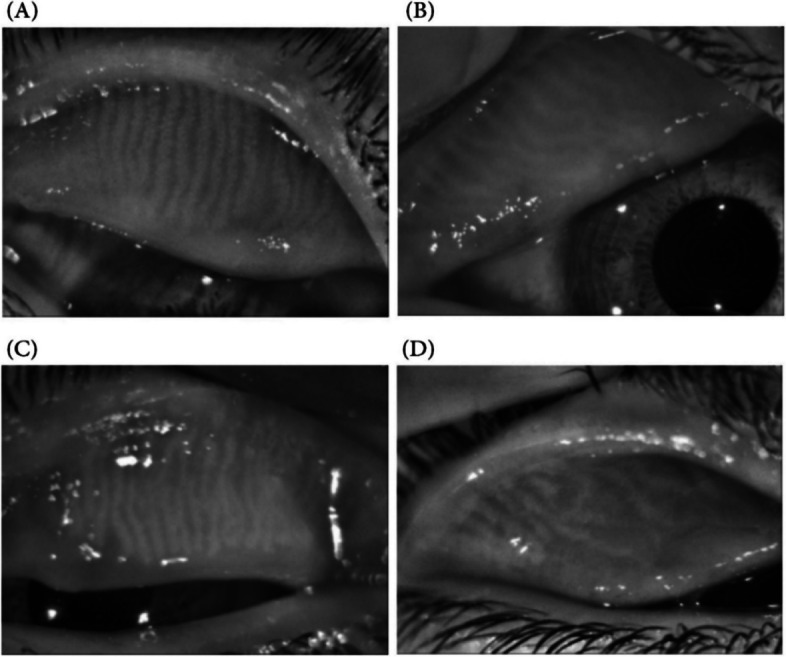
Different grades of Meiboscore: **A**, **B** Low degrees of Meiboscore, **C**, **D** High degrees of meiboscore

Figure 2 shows different grades of meiboscore.

## Discussion

Although RA primarily affects joints, it can affect many organs due to its abnormal systemic immune response, especially the ocular surface manifestation [19, 20]. non-contact Meibography evaluates morphological changes in the meibomian glands, such as distortion, dilation, and dropout. It can reveal detailed changes in meibomian glands (MGs); thus, it is an important tool for diagnosing meibomian glad dysfunction (MGD) and DED [21].

In this study, we measured objectively the degree and extent of dry eye in RA active patients, even if they were asymptomatic, by infrared non-contact meibography in the Scheimpflug topographer (Sirius, CSO, Italy) to examine 60 eyes of 30 subjects with RA. The patients were distributed into two groups. Using the DAS-28 questionnaire, the cases were distributed into the high activity group and low-to-moderate-activity group.

High activity group showed significant affection regarding the mebioscore of the lower and upper lids, total mebioscore, percentage of meibomian glands in upper and lower lids, NIV-BUT of the tear film and NIavg-BUT of the tear film. These findings coincided with Weifang Ma et al. [22] that studied the DED-activity relationship in cases with RA and found that the control group’s TBUT was significantly higher than in the RA group, whereas the meibomian scan was higher, however, RA and DED (*p* > 0.05) did not significantly correlated. Also, Gilbo et al. [23] reported that DED in RA cases had more disease activity. Wolfe and Michaud [24] reported that DED symptoms were higher in RA cases with higher DAS-28, pain, and disability.

Abd-Allah NM et al. [25] concluded that DAS-28 did not significantly correlate with ocular tests for dryness, but RA duration had a significant positive correlation with Schirmer test and ocular staining.

As regards the correlation between DAS-28 and meiboscore, there were moderate correlations between the activity of the disease represented by (DAS-28) and different parameters evaluating eye dryness (meiboscore of the lower and upper lids, total meiboscore, percentage of meibomian glands in upper and lower lids, as well as NIV-BUT and NIavg-BUT of the tear film). These results coincide with Gilbo et al. [23] and Wolfe and Michaud [24]. However, different studies showed different results. For instance, Abd-Allah NM et al. [15], Fujita M [26] concluded that DED should be considered regardless of the RA activity because there is no relation between RA activity and DED severity, Also Zakeri et al. [27] revealed no correlation between DED and RA mean severity.

This study investigated one of the extra-articular manifestations of RA potentially involving the eyes. Common ocular presentations of RA include corneal inflammation, lacrimal gland dysfunction, and uveitis, sharing similar pathogenic mechanisms to DED [28, 29]. It was used in diagnosis and evaluating DED severity and its correlation with RA disease activity and duration, which was found to be a valuable tool as used previously in different diseases [30–33].

### Limitations

There were many limitations in this study, the small number of RA cases and the short duration of follow up, further future studies will be needed with huge number of cases and longer follow up duration.

In conclusion, there was no correlation with duration of RA but moderate correlations between the activity of the disease represented by (DAS-28) and different parameters evaluating eye dryness in RA patients with valuable use of noncontact Meibography to evaluate eye dryness in RA cases. Both Meiboscore and Meiboscale of Non-contact Meibography were found to be useful tools in grading of dry eye disease in different activity grades of Rheumatoid Arthritis.

## Data Availability

All data generated or analyzed during this study are included in this published article.
